# Economic value in the Brain: A meta-analysis of willingness-to-pay using the Becker-DeGroot-Marschak auction

**DOI:** 10.1371/journal.pone.0286969

**Published:** 2023-07-10

**Authors:** Alice Newton-Fenner, Danielle Hewitt, Jessica Henderson, Hannah Roberts, Tyler Mari, Yiquan Gu, Olga Gorelkina, Timo Giesbrecht, Nicolas Fallon, Carl Roberts, Andrej Stancak

**Affiliations:** 1 Department of Psychology, University of Liverpool, Liverpool, United Kingdom; 2 Institute of Risk and Uncertainty, University of Liverpool, Liverpool, United Kingdom; 3 Wellcome Centre for Integrative Neuroimaging, University of Oxford, Oxford, United Kingdom; 4 Henley Business School, University of Reading, Reading, United Kingdom; 5 Management School, University of Liverpool, Liverpool, United Kingdom; 6 Unilever, Research and Development, Port Sunlight, United Kingdom; Juntendo University, JAPAN

## Abstract

Forming and comparing subjective values (SVs) of choice options is a critical stage of decision-making. Previous studies have highlighted a complex network of brain regions involved in this process by utilising a diverse range of tasks and stimuli, varying in economic, hedonic and sensory qualities. However, the heterogeneity of tasks and sensory modalities may systematically confound the set of regions mediating the SVs of goods. To identify and delineate the core brain valuation system involved in processing SV, we utilised the Becker-DeGroot-Marschak (BDM) auction, an incentivised demand-revealing mechanism which quantifies SV through the economic metric of willingness-to-pay (WTP). A coordinate-based activation likelihood estimation meta-analysis analysed twenty-four fMRI studies employing a BDM task (731 participants; 190 foci). Using an additional contrast analysis, we also investigated whether this encoding of SV would be invariant to the concurrency of auction task and fMRI recordings. A fail-safe number analysis was conducted to explore potential publication bias. WTP positively correlated with fMRI-BOLD activations in the left ventromedial prefrontal cortex with a sub-cluster extending into anterior cingulate cortex, bilateral ventral striatum, right dorsolateral prefrontal cortex, right inferior frontal gyrus, and right anterior insula. Contrast analysis identified preferential engagement of the mentalizing-related structures in response to concurrent scanning. Together, our findings offer succinct empirical support for the core structures participating in the formation of SV, separate from the hedonic aspects of reward and evaluated in terms of WTP using BDM, and show the selective involvement of inhibition-related brain structures during active valuation.

## Introduction

In human decision-making, where an individual compares their options and select the course of action with the highest SV, the construction of SV of potential outcomes is critical [1]. Previous theories of decision-making have highlighted rational expectations [2–4] and reference points [5,6] as prominent factors in SV formation. A set of regions have been identified as comprising the brain valuation system, including the ventromedial prefrontal cortex (vmPFC) [7–9], ventral striatum (VS) [10–13], anterior insula (AI) [14–17], posterior parietal cortex (PPC) [18,19], orbitofrontal cortex (OFC) [20–22], amygdala [7,23–26] and anterior cingulate cortex (ACC) [27–30].

Subjective valuation is a complex process requiring the amalgamation of an individual’s perceptions, prior knowledge, and reward expectations of a given stimulus. It has often been implicitly defined through differing methodology; such as liking scales, unpleasantness ratings and binary forced choice decisions in monetary gambling tasks. However, this heterogeneity in methodology can have the consequence of implicitly defining varying conceptualisations of value under the umbrella term of SV, with the potential to conflate SV with other closely related concepts. For example, hedonic understandings of attractiveness [1] and pleasure [31] can be understood as distinct from utilitarian concepts of worth [3] and a willingness to exert effort or a motivation to take on costs [32]. Further, there are established differences in the brain circuitry involved in the liking, wanting, and pleasantness of a reward, in particular in subregions of the VS [33–36]. Whereas finding a reward pleasant or likeable refers to an emotional state and its experiential qualia, the wanting of a reward refers more to the underlying motivational processes and is linked to decision utility [37]. In this way, heterogeneity in the definitions of SV and task paradigms may confound the findings to date, and it is likely that the range of brain regions associated with SV is smaller than indicated by available meta-analyses owing to SV being estimated by hedonic measures. For example, in the interest of maximising the pool of viable studies, Bartra *et al*. (2013) used simple search parameters of “fMRI” AND “reward”. However, as receiving a reward entails multiple other processes in addition to the representation of the SV of the object, such as the pleasantness of positive feedback, the perceived attractiveness of the object, and other hedonic processes, it is not known which part of the brain valuation system would specifically encode SV.

In behavioural economics and neuroeconomics, valuation consolidates multiple determinants of a goods’ value into a singular figure of a given currency. Methodologically, this has several advantages. Firstly, one can assign an economic value to any type of outcome stimulus, such as food, music, pain, or lottery tickets [20,21,38,39]. In this way, experiences in different mediums can all be translated into monetary worth that is subjective to the individual. Secondly, multiple facets of reward receipt, such as the outcomes’ temporal immediacy and probability of reward, can be integrated into a single discounted SV, and so complex options can be compared against each other. Thirdly, economic valuation is applicable to both rewards and punishments: tasks can explore paying for the opportunity to receive a good outcome or to avoid a bad one [40], which allows the relationships between loss and gain to be explored. Fourthly, as monetary scales are linear, the relative relationships of a theoretically infinite number of outcomes can be compared and ranked. Finally, it is intuitive to participants, as individuals are well-versed in weighing up purchasing decisions to maximise their utility in their everyday lives.

WTP is the standard measure of value in economics, and is defined as the maximum amount of currency a customer is willing to part with in order to purchase a product or service. There are several methods that can estimate WTP, either directly or indirectly, and ascertain a consumer’s hypothetical or actual WTP [41]. However, most methods, such as 2 alternative-forced-choice tasks (2AFC), open-ended questions (“what would you be willing to pay for this item?”) or choice-based conjoint analysis (“pick one item from this list of options”) can produce unreliable results [42,43]. This is due to a lack of incentive to induce truth-telling: within the parameters of these mechanisms, participants are not appropriately compensated for revealing the private information of their SVs. Therefore, they may not wish to do so, and the responses may be arbitrarily chosen or due to other motives, reporting SVs that they do not necessarily hold or would act upon. As the participants responses do not hold real consequences, such as a purchasing commitment, their choices may not reflect their true preferences. Consequently, researchers cannot rely on the values participants provide [44,45].

Furthermore, hypothetical purchasing scenarios has been shown to produce consistent behavioural overestimations of WTP in comparison to that of real purchasing scenarios, termed the Hypothetical Bias [46–49]. This effect is strongest in indirect measures, such as in 2AFCs, leading to consistent overestimation of WTP values [50]. Crucially for this work, valuation areas of the brain are also differentially activated by hypothetical and real choices, with greater activity for real purchasing decisions in the orbitofrontal cortex, and conflicting evidence of activation in the ventral striatum for hypothetical choices [51,52].

In contrast, the auction paradigm Becker-DeGroot-Marschak mechanism (BDM), equivalent to a second-price sealed-bid auction, is an incentivized experiment [53]. During a BDM, a player submits a single bid for a given item. Their bid value is compared to a randomly generated price, and if the player’s bid exceeds or equals this price they win the item and pay the random price. If the player’s bid does not exceed that of the random number generator, they win nothing and lose nothing. As the player’s bid value is used to produce the outcome directly affecting the player, bidding one’s true SV is the dominant strategy. If the player underbids, they only risk not winning the item for a price that they would be willing to pay, and if the player overbids, they only risk winning the item for more than they are willing to pay. In this way, their bid value can also be thought of as their reservation price, or indifference point [54]. Formal proof of the dominant strategy in BDM Auctions can be seen in Supporting information.

The present study proposed to compare brain activations associated with SV as defined by WTP through a BDM by employing a coordinate based meta-analysis with activation likelihood estimation (ALE) [55,56]. A single paradigm was utilised, therefore avoiding the confounding effects of task heterogeneity. The BDM has become increasingly popular in neuroeconomics in recent years, in no small part to its use in the seminal paper by Plassmann, O’Doherty and Rangel (2007), so that there now exists a sufficient body of work to conduct a meta-analysis of fMRI studies evaluating WTP using the BDM.

Activation in the brain valuation system tends to increase when considering the SV of the available options during choice, as well as with the value of the reward received, and responds to both primary and secondary forms of reward [17,57,58]. This suggests that a domain-general system in the brain is responsible for the encoding of SV across multiple decision stages and reward types [59]. Furthermore, evidence for automaticity in value attribution has been provided in a number of previous studies [7,60–62]. For instance, the brain valuation system scales the SV of objects even if participants are asked to make value-irrelevant judgements, such as perceptual discernment of stimuli characteristics [60,61,63]. To investigate automaticity of subjective valuation, we also compared the WTP contrasts in studies for which WTP was elicited during fMRI scanning (concurrently) or outside of the scanner (consecutively). We posited that the brain regions encoding WTP would be invariant to the concurrency of the BDM auction session and fMRI recording, as the WTP values would be automatically invoked even in non-incentivized tasks or during the passive viewing of objects even in absence of choice selection.

## Methods

An a priori protocol for this meta-analysis was preregistered at The Open Science Framework: https://osf.io/vpt3d.

### Information sources and search strategy

The formal search strategy consisted of systematically examining 3 electronic databases (PubMed, Scopus, PsycINFO) through August 2022 using the MeSH search terms (fMRI OR functional magnetic resonance imaging OR neuroimaging) AND (willing to pay OR willing-to-pay OR willingness to pay OR willingness-to-pay OR WTP OR BDM OR Becker–DeGroot–Marschak OR Becker DeGroot Marschak OR economic valuation). Searches were restricted to terms found in the title or abstract of the articles. No date limit was set for the searches.

During the search process, the authors noticed that several potentially eligible papers did not refer to the task as a BDM auction; for example, one article in the final corpus cites Plassmann, O’Doherty and Rangel (2007) and not Becker, DeGroot and Marschak (1964) as the task originators [64]. Therefore, for completeness, a comprehensive manual search of the reference sections and citation lists of identified articles was conducted to supplement the formal searches. Previous meta-analyses of fMRI studies on human reward [16,65–67] were also screened for additional articles.

### Article selection and extraction of data

Formal database searches were conducted by ANF, as were supplementary and manual searches. One author (ANF) was responsible for assessment of articles for inclusion, with three authors (AS, JH and DH) conducting 2^nd^ reviews of 10% of the collected articles each (totalling 30% of the initially identified articles). Decisions regarding final article inclusion were determined by discussion. One author (ANF) extracted the relevant coordinate data, and these were cross-checked by a second author (CR).

### Eligibility criteria

The criteria for inclusion were 1) any human fMRI studies published through to August 2022; 2) original English language articles; 3) published in a peer-reviewed journal; 4) used a Becker-DeGroot-Marschak task to elicit WTP; 5) computed the correlation of Blood Oxygenation Level Dependent (BOLD) activity to the WTP value; 6) coordinates were reported in the article or supplementary material in Montreal Neurological Institute (MNI) [68] or Talairach space [69]; 7) data were obtained from a healthy population (systemic disease-free); 8) whole-brain analysis were reported with thresholding of (or equivalent to) *p* < 0.001 uncorrected voxelwise throughout the whole brain with at least *p* < 0.05 cluster level correction (or equivalent) declared [70].

### Additional handling of data

We excluded papers which only reported region of interest (ROI) analysis, which may bias results towards more established or accepted regions [71]. One of the studies in the final sample, Chib et al. (2009), reported three separate activation maps for the computation of WTP for three different categories of goods: money, trinkets and snacks. In the interest of including a wide variety of stimuli, the activation map for trinkets was selected for inclusion in the meta-analysis. Studies that reported coordinates in Talariach space were converted into MNI coordinates using GingerALE (Brainmap GingerALE version 3.0.2; Research Imaging Institute; http://brainmap.org) [72].

### Activation likelihood estimation meta-analysis

A primary ALE meta-analysis was conducted for experiments using BDM paradigms to elicit WTP measures, contrasting increasing WTP with increasing BOLD responses. Decreasing activations in line with increasing WTP were not investigated. See Table 1 for data on the included studies. Subsequently, an exploratory secondary analysis was performed on the same dataset, split by concurrency of BDM task with fMRI scanning, with 16 BDM tasks performed inside the scanner (concurrently) and 8 BDM tasks performed outside the fMRI scanner (consecutively).

**Table 1:** Studies and experiments included in ALE meta-analyses on willingness-to-pay in human adults.

Authors	Year	Title	N (men)	Mean age (SD)	Concurrency of recordings	Main Findings
**Chib *et al. [73]***	2009	Evidence for a common representation of decision values for dissimilar goods in human ventromedial prefrontal cortex	32 (25)	23	Consecutive	Common currency mechanism for decision, outcome and anticipatory values encoded in the vmPFC
**De Martino *et al. [21]***	2009	The neurobiology of reference-dependent value computation	18 (10)	22.2 (3.1)	Consecutive	OFC and dorsal striatum encoded absolute WTP, VS indexed endowment effect
**De Martino *et al. [74]***	2013	Confidence in value-based choice	20 (NA)	24.24	Consecutive	VmPFC encodes SV comparisons and subjective confidence in decisions
**Enax *et al. [75]***	2015	Nutrition labels influence value computation of food products in the ventromedial prefrontal cortex	25 (11)	23.3 (4.4)	Concurrent	VmPFC, ACC, caudate nucleus and putamen encode WTP. vmPFC modulated by the inferior frontal gyrus / dorsolateral prefrontal cortex (dlPFC) when rating unhealthy foods, and by the posterior cingulate cortex (PCC) when rating healthy foods
**Gluth *et al. [76]***	2015	Effective Connectivity between Hippocampus and Ventromedial Prefrontal Cortex Controls Preferential Choices from Memory	30 (12)	26.1 (3.9)	Consecutive	VS, vmPFC and hippocampus encode the value of the chosen option, vmPFC encodes the value of the unchosen option
**Grueschow *et al. [61]***	2015	Automatic versus Choice-Dependent Value Representations in the Human Brain	26 (13)	RG 20–28	Consecutive	Medial PFC and VS activity correlated with SVs during purchasing but not perceptual decisions. PCC activity correlated with both
**Hare *et al. [77]***	2008	Dissociating the role of the orbitofrontal cortex and the striatum in the computation of goal values and prediction errors	16 (9)	24.1, RG 19–38	Consecutive	Goal values correlated with medial OFC activity, decision values correlated with central OFC activity, and prediction errors correlated with VS activity
**Hutcherson *et al. [78]***	2012	Cognitive regulation during decision making shifts behavioral control between ventromedial and dorsolateral prefrontal value systems	26 (17)	22, RG 19–28	Concurrent	VmPFC and dlPFC correlated with WTP, indulging upregulated vmPFC signals, behavioural control modulation increased dlPFC contribution
**Janowski *et al. [79]***	2013	Empathic choice involves vmPFC value signals that are modulated by social processing implemented in IPL	32 (32)	22.8 (3.9)	Concurrent	Playing in a BDM for others engages vmPFC, modulated by activity from inferior parietal lobule (IPL)
**Linder *et al. [80]***	2010	Organic labeling influences food valuation and choice	30 (15)	26.03, RG 23–38	Concurrent	Activity in VS increased with WTP for organic foods
**Mackey *et al. [81]***	2016	Greater preference consistency during the Willingness-to-Pay task is related to higher resting state connectivity between the ventromedial prefrontal cortex and the ventral striatum	19 (9)	31.5 (11)	Concurrent	Ventral precuneus, vmPFC and PCC activity increased with WTP
**McNamee *et al. [82]***	2013	Category-dependent and category-independent goal-value codes in human ventromedial prefrontal cortex	13 (8)	22.1 (3.6)	Concurrent	Medial PFC implements a goal-value code independent of stimulus category, medial OFC and vmPFC contain category dependent value codes
**Medic *et al. [83]***	2014	Dopamine modulates the neural representation of subjective value of food in hungry subjects	47 (23)	23.8 (3.2)	Concurrent	Infusion of dopamine agonist increased the inferior parietal gyrus/intraparietal sulcus response to WTP
**Merchant *et al. [84]***	2020	Neural Substrates of Food Valuation and Its Relationship With BMI and Healthy Eating in Higher BMI Individuals	93 (16)	39.25 (3.5)	Concurrent	vmPFC, anterior VS, bilateral AI, and the ACC activity correlated with WTP, vmPFC activity linked to valuation of healthy (vs unhealthy) items
**Motoki *et al. [85]***	2019	Common neural value representations of hedonic and utilitarian products in the ventral stratum: An fMRI study	27 (21)	20.37 (1.15)	Concurrent	Values of hedonic and utilitarian goods are similarly processed in the VS during BDM
**Plassmann *et al. [86]***	2010	Appetitive and aversive goal values are encoded in the medial orbitofrontal cortex at the time of decision making	20 (15)	23.25, RG 19–34	Concurrent	Medial OFC and the dlPFC correlated with appetitive and aversive goal values
**Plassmann *et al. [20]***	2007	Orbitofrontal cortex encodes willingness to pay in everyday economic transactions	19 (16)	25.45, RG 18–46	Concurrent	Medial OFC and the dlPFC correlated with WTP
**Rihm *et al. [87]***	2019	Sleep deprivation selectively upregulates an amygdala–hypothalamic circuit involved in food reward	32 (32)	26.13 (3.8)	Consecutive	WTP increased when sleep deprived. Upregulation of hypothalamic valuation signals and amygdala–hypothalamic coupling after sleep deprivation
**Seak *et al. [88]***	2021	Single-Dimensional Human Brain Signals for Two-Dimensional Economic Choice Options	24 (11)	25.4, RG 19–36	Concurrent	Activity in striatum, midbrain, and OFC correlated with revealed preference across choice indifference curves
**Setton *et al. [89]***	2019	Mind the gap: Congruence between present and future motivational states shapes prospective decisions	25 (10)	22.52 (2.79) RG 18–30	Concurrent	VS activity positively correlated with level of prospection bias towards food items
**Tang *et al. [90]***	2014	Behavioral and neural valuation of foods is driven by implicit knowledge of caloric content	29 (NA)	(NA)	Concurrent	Activity in the vmPFC linked with caloric density of auction food items
**Verdejo ‐Román *et al. [91]***	2017	Brain reward system’s alterations in response to food and monetary stimuli in overweight and obese individuals	81 (38)	33.35 (6.28)	Concurrent	Obese group showed greater activation in VS and dorsal striatum than overweight and normal weight groups
**Waskow *et al. [92]***	2016	Pay what you want! A pilot study on neural correlates of voluntary payments for music	25 (13)	35.08 (17.71)	Concurrent	Compared “Pay What You Want” (PWYW) to fixed price condition of BDM. OFC, medial PFC and ACC activity correlates with WTP in BDM, no correlation for PWYW found
**Zangemeister *et al. [93]***	2019	Neural activity in human ventromedial prefrontal cortex reflecting the intention to save reward	22 (NA)	NA	Consecutive	vmPFC activity correlates with value and one’s intention to save during sequential economic choices

To determine consistency in reported regions of neural activation, for our primary analysis we conducted a coordinate-based ALE meta-analysis (single dataset analysis). The analysis was performed using Brainmap GingerALE version 3.0.2. Standardized procedures and default parameters for performing ALE using GingerALE were followed, as outlined in the GingerALE user manual (Research Imaging Institute; http://brainmap.org) and Eickhoff et al. (2016).

The concordance of ALE values throughout the brain for WTP were evaluated in comparison to random distributions using permutation analysis [94] with 10,000 permutations. An initial cluster forming threshold (uncorrected *p* < .001) was implemented followed by cluster-level Family-wise error (FWE) correction (*p* < .05) to identify relevant ALE regions as previously recommended [71,95]. Multi-image analysis GUI (http://ric.uthscsa.edu/mango) was used to overlay ALE maps onto an anatomical image using MNI coordinates.

Resulting ALE maps for WTP for concurrency of BDM task were compared using conjunction and contrast analyses. The same protocol as previous ALE meta-analyses conducted in our lab was followed [96,97]. Again, permutation analysis was first performed on the concurrent/consecutive sub-groups with 10,000 permutations, an initial cluster forming threshold (uncorrected *p* < .001) and a cluster-level Family-wise error (FWE) correction of *p* < .05. For cluster analysis, an uncorrected threshold of p < 0.05 and a minimum cluster size of 200 mm^3^ was adopted as previously recommended [72,95,98–100].

To facilitate future research, ROIs created using the resultant unthresholded meta-analytic clusters are available via NeuroVault (https://neurovault.org/collections/IBLCLBYH/images/785459/).

### Fail-safe N analysis

Co-ordinate-based meta-analyses can be affected by publication bias, where unpublished null results may alter or invalidate findings: known as the “file drawer problem” [101,102]. The fail-safe N (FSN) analysis addresses this issue, assessing the robustness of ALE clusters by introducing null pseudo-studies as noise to the ALE cohort to calculate the amount of contra-evidence that the ALE can tolerate [103]. It is posited that the number of unpublished fMRI studies is lower than behavioural studies due to their greater expense and time-demands. Recent estimations propose that for every 100 published fMRI studies, there are between 6–30 unpublished studies which report no local maxima [104]. Using the upper bound, an estimate for the number of unpublished WTP studies using BDM used in the FSN analysis (minimum FSN) was set at 7 null pseudo-studies [105]. Further, to ensure that no single study is driving the ALE scores of each cluster, a maximum FSN was set at 146, requiring at least a 10% contribution from the cohort studies [95].

## Results

**Fig 1:**
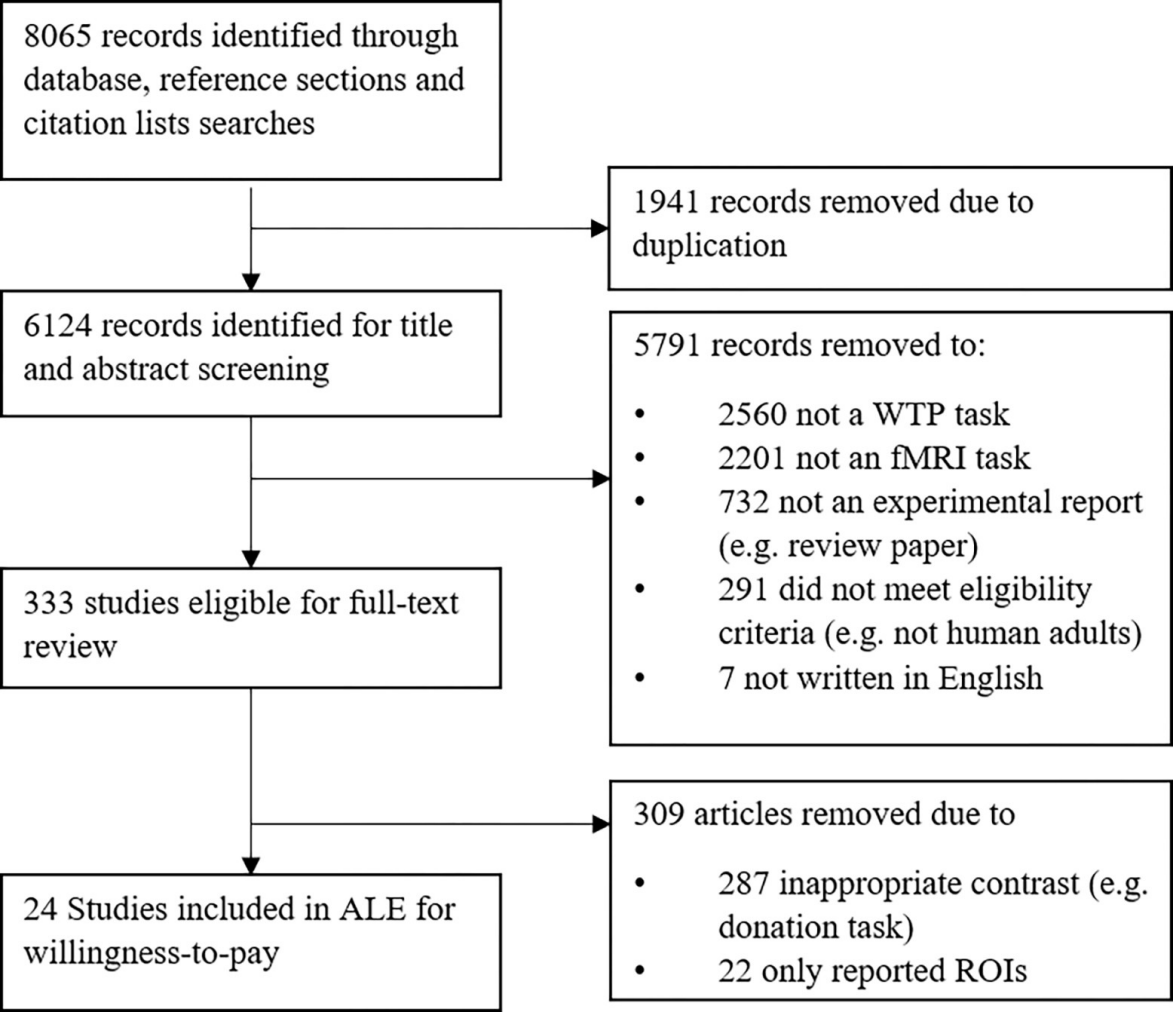
Flow chart outlining the formal search and eligibility screening process.

Fig 1 illustrates a flowchart indicating the study selection steps. A total of 8065 records were returned from initial searches. Of these, 1940 were duplicates from repeated searches and removed in the first step. A further 5791 articles were removed following the initial review of titles and abstracts. Studies excluded at this stage included: those that were not reported in English (7) those where it was clear and obvious that no suitable (i.e. healthy, human adult) population was reported (291), where it was clear and obvious that they did not utilize a WTP task (2560), not an experimental report (e.g. review articles) (732), not fMRI method (2201). Furthermore, following full-text review a further 309 articles were removed including those which exhibited an inappropriate contrast (e.g. donation task) (287), or which only reported ROI analyses (22), leaving a total of 24 studies for the analyses of WTP (Table 1).

### Significant ALE clusters for WTP

**Fig 2:**
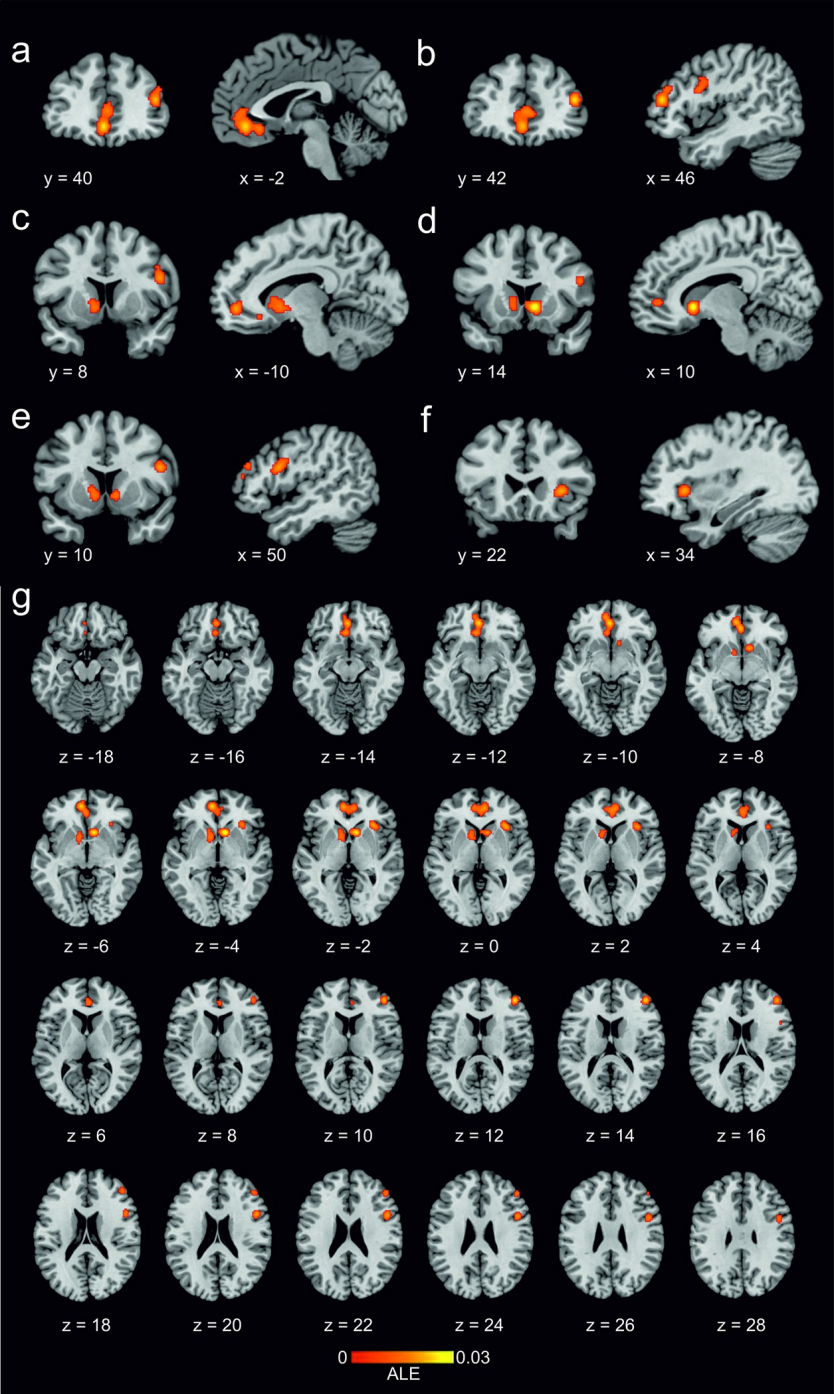
The location of significant ALE clusters from the meta-*analysis* of concordant activations for WTP. A–F show coronal and sagittal slices at the cluster peak in: (A) vmPFC with sub-cluster in the ACC, (B) right dlPFC, (C) left VS, (D) right VS, (E) right IFG and (F) right AI. (G) shows all clusters in axial orientation. Results are displayed overlaid onto a standardized MNI template anatomical brain. ALE scores are indicated by the colour bar.

The WTP ALE meta-analysis pooled data from a total of 731 participants and 190 reported foci from the 24 studies. The results (see Table 2) revealed six significant clusters, where ALE values represent consistent spatial activations which increased in line with WTP. The largest cluster was elicited in the vmPFC (Brodmann areas 10 and 32) centring on the medial prefrontal gyrus and extending into the left subgenual ACC (sgACC, Brodmann area 32) and right pregenual ACC (pgACC, Brodmann areas 24 and 32). Further clusters were found encompassing the bilateral VS, in the right dorsolateral prefrontal cortex (dlPFC) (Brodmann areas 45 and 46), the right inferior frontal gyrus (IFG) (Brodmann area 44) and the right AI (Brodmann area 13). We found satisfactory robustness of our results against publication bias, with all but the right AI cluster showing an FSN above the minimum imposed, indicating an overall robust convergence of foci. Fig 2 illustrates the location of significant ALE clusters from the meta-analysis of WTP.

**Table 2:** Locations of significant clusters from the ALE map of WTP.

*Cluster*	*Label*	*Volume (mm* ^*3*^ *)*	*# Studies* *(foci)*	*ALE peak*	*Brodmann area*	*MNI co-ordinates (x*, *y*, *z)*	*Talairach co-ordinates (x*, *y*, *z)*
1	vmPFC L	4584	17 (19)	0.02463	10/32	-2, 40, -12	-2, 35, -12
	vmPFC L			0.02412	10/32	-8, 48, -6	-8, 43, -6
	Subgenual ACC L			0.01898	32	-4, 28, -12	-4, 24, -10
	Pregenual ACC R			0.01955	10/32	6, 46, 0	5, 41, 0
2	dlPFC R	1072	5	0.02479	45/46	46, 42, 12	45, 41, 13
	dlPFC R			0.01652	45/46	48, 38, 22	47, 38, 22
3	VS L	1056	5	0.01670	n/a	-10, 8, -4	-10, 5, 0
4	VS R	1008	4 (5)	0.02956	n/a	10, 14, -4	9, 11, 0
5	IFG R	968	6	0.01982	44	50, 10, 20	48, 9, 21
6	AI R	784	4	0.02132	13	34, 22, 0	32, 19, 3

L, left hemisphere; R, right hemisphere.

#### Contrast and conjunction analyses

**Fig 3:**
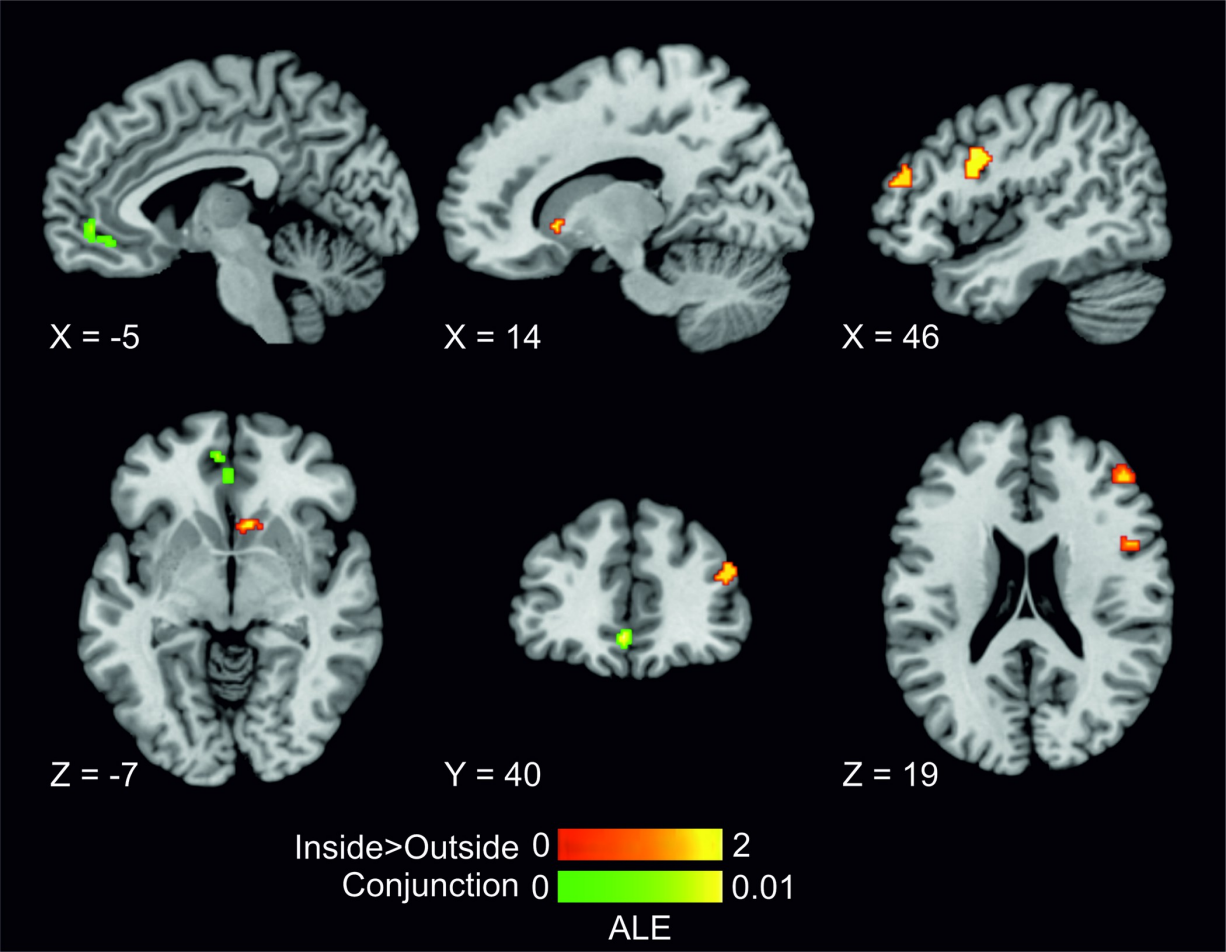
The location of significant clusters from conjunction and contrast analyses of ALE maps for concurrent (inside) and consecutive (outside) recordings. Results are displayed overlaid onto standardized MNI template anatomical brain in as a montage of sagittal, coronal and axial slices through the clusters. ALE scores are indicated by the colour bars.

To investigate to what extent the relationship between brain activation and reported WTP is automatically engaged, a contrast analysis was conducted comparing the ALE maps of concordant activations for concurrency of BDM performance and fMRI recording. Data was pooled from the entire cohort of 24 studies, with a total of 16 studies (535 participants and 158 reported foci) for concurrent recording and 8 studies (196 participants and 32 reported foci) for consecutive recordings. The contrast analysis revealed 3 clusters indicative of increased activation likelihood estimates for concurrent scanning relative to consecutive scanning. These regions were in the right IFG, right dlPFC and right caudate (Table 3, Fig 3).

**Table 3:** Locations of significant clusters from conjunction and contrast analyses of WTP for concurrent and consecutive recordings.

Cluster	Label	Volume (mm^3^)	ALE peak	Brodmann area	MNI co-ordinates (x, y, z)	Talairach co-ordinates (x, y, z)
**Conjunction Analysis**						
1	OFC L	192	0.0100	11	-2, 40, -10	-2, 35, -10
2	vmPFC L	104	0.0096	10/32	-6, 50, -4	-6, 44, -5
**Contrast Analysis–Concurrent > Consecutive**						
1	IFG R	864	0.0173	6	43, 4, 31	42, 4, 31
	IFG R		0.0328	44	45, 8, 26	43, 8, 26
	IFG R		0.0328	44	50, 6, 24	48, 5, 25
2	dlPFC R	336	0.0333	10	46, 45, 16	45, 45, 16
	dlPFC R		0.0494	10	46, 40, 20	45, 40, 20
3	Caudate R	272	0.0246	n/a	14, 18, -4	13, 15, 0
	Subgenual ACC R		0.025	25	4, 18, -4	3, 15, -1
	Caudate R		0.0265	n/a	10, 18, -6	9, 15, -2

L, left hemisphere; R, right hemisphere.

Additionally, given the likelihood of an extended network of reward processing, a conjunction analysis was conducted to establish commonalities in activation profiles between the two types of recording. The results highlighted an overlap of activation likelihood coordinates in two clusters, in the left vmPFC and the left OFC (Table 3, Fig 3).

## Discussion

Performing subjective valuation judgements, and carrying out choices based on these valuations, is an integral part of everyday life. In no case is this more pertinent than in economic purchasing decisions. The present meta-analysis was conducted to identify the core brain valuation system subserving computation of SV as determined by an incentive-compatible WTP metric. The primary ALE analysis identified the locations of positive effects of SV on BOLD activity, where positive effects elicited larger BOLD responses increasing with WTP. The largest concordant activation to WTP was located in the left vmPFC, with a sub-cluster of activation extending into the right pgACC and left sgACC. Additionally, the bilateral VS, right dlPFC, right IFG and right AI also demonstrated significant levels of consistent spatial activation for WTP. Secondary contrast and conjunction analysis established distinct and overlapping neural substrates underlying value-related activations according to concurrency of BDM and fMRI recordings, contrary to our hypothesis. As the pool of studies used a wide range of stimuli types, this analysis shows that the regions elicited play a central role in the encoding of decision values in a wide number of economic settings. Critically, by using an experimental design that allowed us to identify areas that encode for WTP, we were able to isolate those involved in economic choice from other areas that are related to hedonic aspects such as arousal or familiarity.

The results from this meta-analysis confirm the vmPFC as a core brain area of SV computation, with 71% of the pool of studies contributing to the vmPFC cluster in the main analysis. Notably, activations in vmPFC and bilateral striatum are in good agreement with a previous fMRI meta-analysis [16] which highlighted these regions, alongside the PCC, ACC, pre-supplementary motor area and insula, as parts of the brain valuation system. The role of vmPFC in the construction of SV also corroborates with positron-emission tomography studies [106], as well as single-cell recordings [107], lesion [108,109] and animal studies [110,111]. Further, our conjunction analysis showed that the vmPFC is the only region to display consistent spatial activation regardless of concurrency of explicit valuation responses and fMRI recording. This suggests that the vmPFC may be the principal region responsible for SV processing in the brain.

The activation shown in the vmPFC extended into the rostral portions of the ACC. Typically, ACC activations are linked to emotions [112,113]; resting-state fMRI studies show that the ACC is most functionally connected with areas implicated in affective processing, with pgACC having more widespread connections than sgACC [114]. Both the pgACC and sgACC have also been shown to be modulated by an overestimation of probabilities of good outcomes [115], and sgACC activity in particular positively correlates with expected value of an outcome [116,117]. Further to this, ACC neurons in non-human primates encode the values of the chosen options during decision-making [118–120]. It may be that activity found in the ACC is due to the uncertainty implicit in the BDM, with the risk of good and bad outcomes being directly linked to the participant’s expressed expected values.

The VS is also frequently cited as a primary region of reward processing [67,77,121,122]. Both the vmPFC and striatum are key dopaminergic areas, receiving dopaminergic projections from the midbrain [123], and are well established to be involved in option valuation and comparison [124–126]. Single-cell recordings in rhesus macaques show extensive similarities in neuron firing patterns in the VS and vmPFC during risky reward-based choice [121]. Activity in the VS has been shown to be mediated by the magnitude of expected reward in both humans [106,127,128] and non-human primates [121,129]. Our findings confirm that the vmPFC and VS have signals that are directionally related to SV in a similar way as they both scale in activity with WTP.

The present meta-analysis also showed the right AI was consistently activated by SV. This brain region has considerable functional heterogeneity, being involved in a wide variety of functions such as interoception [130,131], emotion processing [132,133] and arousal [134]. With regards to reward processing, the AI is extensively connected to dopaminergic regions such as the vmPFC, amygdala and ventral striatum [135], and is implicated in loss prediction [136], aesthetic appraisal [137] and in economic uncertainty [123,138,139]. The AI has been proposed as a candidate for generalized uncertainty processing, as the perception of risk and uncertainty involves integrating both external probability computation and the internal qualia of emotions [17,140]. Our findings support this hypothesis, as the parameters of the BDM are such that players are in a situation of static risk: players are presented with potential economic losses if they overbid (see the winner’s curse) [141,142] and an increase in likelihood of a social loss if they underbid (in the form of negative feedback such as “you lose”).

The delineation of activation patterns between concurrent and consecutive execution of task and fMRI scanning in the current context is related to the concepts of task relevance, and the automaticity of value processing [7,60,61]. In line with previous studies demonstrating task-irrelevant underlying value-related neural computations, we hypothesised that areas of the brain valuation system would be activated in proportion to WTP regardless of the task being performed in the scanner. However, activation in the right dlPFC and IFG scaled with WTP and also showed preferential activation in concurrent over consecutive scanning. Both the dlPFC and IFG are known to be central to executive functioning, attention and cognitive control [41,143–148]. Previous work has linked the dlPFC to behavioural restraint and delayed reward [149], demonstrating that individuals who successfully inhibit their value responses during self-control tasks exhibit greater dlPFC activity than those who did not [148,150]. The IFG is involved in the overweighting of private vs public information [151] and conflict resolution [152] during decision-making. While not being integral members of the brain valuation system, such as that described by Bartra et al. (2013), the dlPFC/IFG may instead modulate valuation activity in the vmPFC to induce behavioural restraint [149,153]. This is supported by the contrast analysis, as the dlPFC/IFG would only be engaged during active bidding and not non-incentivised tasks or passive viewing. It is possible that during the BDM, the dlPFC/IFG acts as a self-control mechanism interacting with the valuation system to optimise bidding outcomes [148].

As noted earlier, previous investigation has found a large network of brain areas involved in the formation and updating of subjective valuation [16,67]. To this point, a key finding of this meta-analysis is the notable absence of some of these areas in the patterns of consistent activation. For instance, we found no correlation with WTP in the PPC or the amygdala, both of which have been implicated in reward processing [24,154,155]. Most notably, previous fMRI meta-analyses of SV using other tasks have found larger clusters in the vmPFC incorporating the medial OFC [59,77,156], whereas the vmPFC cluster found in our main analysis did not. Neural activation in the OFC has been consistently linked to subjective pleasantness of various stimuli [see supplementary materials of 117 for review]. The delineation of SV of an object from its hedonic pleasure in the present meta-analysis suggests that the OFC may be involved with evaluation of subjective liking as opposed to WTP [157].

The present study is not without its limitations. It should be acknowledged that the BDM has been found to be not incentive compatible in certain circumstances, such as when the object being valued is a lottery [158]. Furthermore, there is evidence that bid values in second-price sealed bid auctions can be impacted by subjective perceptions of uncertainty [159] and social competition [160]. Furthermore, the decision to focus on the BDM task, while allowing a clean analysis of SV computation without the confounding effects of task heterogeneity, resulted in a smaller final cohort. This meta-analysis exceeded the recommendation of at least 17 independent studies for ALE analysis in order to be confident that the results are not biased by any individual experiment from the cohort [95]. However, due to the subsequent split into two subgroups for recording concurrency, it may be premature to draw strong conclusions from the secondary contrast and conjunction analysis. These preliminary distinctions between the effects of concurrency of recordings on SV representation would benefit from clarification by more, higher powered experiments. This would also afford the opportunity to better disentangle any neural differences between passive viewing, binary choice and bid value activation patterns. Here, the aim was to focus on concordance of activations across studies which utilized whole-brain analyses and robust statistical thresholding to reveal the core regions of the brain which demonstrate subjective valuation activations regardless of existing bias. Permitting less stringent search methods would have been detrimental to the integrity of the present investigation. Many other WTP tasks are not sufficiently incentivised, and therefore the WTP values are not reliable indicators of SV [44,92]. We should also note that all but one of the clusters (right AI) in the main analysis passed the FSN analysis for potential publication bias, indicating their stability. With the growing popularity of the BDM, a follow up investigation utilizing a larger cohort would further enhance the robustness of these results.

To conclude, we used ALE analyses to map consistent patterns of cerebral activations involved in SV as determined by the behavioural-economic tool of BDM, which pinpoints SV as WTP. The findings document both overlap and dissociations of valuation regions engaged by concurrency of task and scanning. The BDM paradigm has the ability to differentiate economic value from other factors that contribute towards subjective valuation, such as emotional processing, autonomic responses, associative learning, perceptual attention and motor control. We believe that the present meta-analysis represents the most succinct evidence to date of the core brain regions that encode consumers’ economic valuations of goods. Knowledge of the distinct and overlapping roles of these brain areas offers unique insights for both theoretical and applied neuroeconomic research.

## Supporting information

S1 File Formal proof of the dominant strategy in BDM auctions. (DOCX)

S2 File Literature search and final dataset with co-ordinates for ALE analysis. (XLSX)

S3 File Completed PRISMA checklist for meta-analysis. (DOCX)

## References

[1] Rangel A., Camerer C., and Montague P.R., A framework for studying the neurobiology of value-based decision making. Nat Rev Neurosci, 2008. 9(7): p. 545–56. doi: 10.1038/nrn2357 18545266 PMC4332708

[2] Mongin P., Expected utility theory. 1998.

[3] Moscati I., Measuring utility: From the marginal revolution to behavioral economics. 2018: Oxford Studies in History of E.

[4] Von Neumann J. and Morgenstern O., Theory of games and economic behavior. 2007: Princeton university press.

[5] Tversky A. and Kahneman D. Rational choice and the framing of decisions. 1989. Berlin, Heidelberg: Springer Berlin Heidelberg.

[6] Kahneman D. and Tversky A., Choices, values, and frames. Handbook of the Fundamentals of Financial Decision Making: Part I, 2013: p. 269–278.

[7] Lebreton M., et al., An automatic valuation system in the human brain: evidence from functional neuroimaging. Neuron, 2009. 64(3): p. 431–9. doi: 10.1016/j.neuron.2009.09.040 19914190

[8] Winecoff A., et al., Ventromedial Prefrontal Cortex Encodes Emotional Value. The Journal of Neuroscience, 2013. 33(27): p. 11032–11039. doi: 10.1523/JNEUROSCI.4317-12.2013 23825408 PMC3718369

[9] Kim S., Hwang J., and Lee D., Prefrontal Coding of Temporally Discounted Values during Intertemporal Choice. Neuron, 2008. 59(1): p. 161–172. doi: 10.1016/j.neuron.2008.05.010 18614037 PMC2593737

[10] Peters J. and Buchel C., Overlapping and distinct neural systems code for subjective value during intertemporal and risky decision making. J Neurosci, 2009. 29(50): p. 15727–34. doi: 10.1523/JNEUROSCI.3489-09.2009 20016088 PMC6666169

[11] Levy I., et al., Neural Representation of Subjective Value Under Risk and Ambiguity. Journal of Neurophysiology, 2010. 103(2): p. 1036–1047. doi: 10.1152/jn.00853.2009 20032238

[12] Delgado M.R., et al., Dorsal striatum responses to reward and punishment: Effects of valence and magnitude manipulations. Cognitive, Affective, & Behavioral Neuroscience, 2003. 3(1): p. 27–38. doi: 10.3758/cabn.3.1.27 12822596

[13] Delgado M.R., et al., Tracking the Hemodynamic Responses to Reward and Punishment in the Striatum. Journal of Neurophysiology, 2000. 84(6): p. 3072–3077. doi: 10.1152/jn.2000.84.6.3072 11110834

[14] Kuhnen C.M. and Knutson B., The neural basis of financial risk taking. Neuron, 2005. 47(5): p. 763–770. doi: 10.1016/j.neuron.2005.08.008 16129404

[15] Knutson B., et al., Neural predictors of purchases. Neuron, 2007. 53(1): p. 147–56. doi: 10.1016/j.neuron.2006.11.010 17196537 PMC1876732

[16] Bartra O., McGuire J.T., and Kable J.W., The valuation system: a coordinate-based meta-analysis of BOLD fMRI experiments examining neural correlates of subjective value. NeuroImage, 2013. 76: p. 412–27. doi: 10.1016/j.neuroimage.2013.02.063 23507394 PMC3756836

[17] Sescousse, et al., Processing of primary and secondary rewards: A quantitative meta-analysis and review of human functional neuroimaging studies. Neuroscience & Biobehavioral Reviews, 2013. 37(4): p. 681–696. doi: 10.1016/j.neubiorev.2013.02.002 23415703

[18] Platt M.L. and Glimcher P.W., Neural correlates of decision variables in parietal cortex. Nature, 1999. 400(6741): p. 233. doi: 10.1038/22268 10421364

[19] Glimcher P.W., The neurobiology of visual-saccadic decision making. Annu Rev Neurosci, 2003. 26: p. 133–79. doi: 10.1146/annurev.neuro.26.010302.081134 14527268

[20] Plassmann H., O’Doherty J., and Rangel A., Orbitofrontal cortex encodes willingness to pay in everyday economic transactions. J Neurosci, 2007. 27(37): p. 9984–8. doi: 10.1523/JNEUROSCI.2131-07.2007 17855612 PMC6672655

[21] De Martino B., et al., The neurobiology of reference-dependent value computation. J Neurosci, 2009. 29(12): p. 3833–42. doi: 10.1523/JNEUROSCI.4832-08.2009 19321780 PMC2722101

[22] Padoa-Schioppa C. and Assad J.A., Neurons in the orbitofrontal cortex encode economic value. Nature, 2006. 441(7090): p. 223–226. doi: 10.1038/nature04676 16633341 PMC2630027

[23] Basten U., et al., How the brain integrates costs and benefits during decision making. Proc Natl Acad Sci U S A, 2010. 107(50): p. 21767–72. doi: 10.1073/pnas.0908104107 21118983 PMC3003102

[24] De Martino B., Camerer C.F., and Adolphs R., Amygdala damage eliminates monetary loss aversion. Proc Natl Acad Sci U S A, 2010. 107(8): p. 3788–92. doi: 10.1073/pnas.0910230107 20142490 PMC2840433

[25] Cardinal R.N., et al., Emotion and motivation: the role of the amygdala, ventral striatum, and prefrontal cortex. Neuroscience & Biobehavioral Reviews, 2002. 26(3): p. 321–352. doi: 10.1016/s0149-7634(02)00007-6 12034134

[26] Holland P.C. and Gallagher M., Amygdala–frontal interactions and reward expectancy. Current Opinion in Neurobiology, 2004. 14(2): p. 148–155. doi: 10.1016/j.conb.2004.03.007 15082318

[27] Walton M.E., et al., Adaptive decision making and value in the anterior cingulate cortex. NeuroImage, 2007. 36: p. T142–T154. doi: 10.1016/j.neuroimage.2007.03.029 17499161 PMC2954047

[28] Botvinick M.M., et al., Conflict monitoring and cognitive control. Psychological Review, 2001. 108(3): p. 624–652. doi: 10.1037/0033-295x.108.3.624 11488380

[29] Shenhav A., Cohen J.D., and Botvinick M.M., Dorsal anterior cingulate cortex and the value of control. Nature Neuroscience, 2016. 19(10): p. 1286–1291. doi: 10.1038/nn.4384 27669989

[30] Vassena E., Deraeve J., and Alexander W.H., Surprise, value and control in anterior cingulate cortex during speeded decision-making. Nature Human Behaviour, 2020. 4(4): p. 412–422. doi: 10.1038/s41562-019-0801-5 31932692

[31] Jiang T., et al., Reward for food odors: an fMRI study of liking and wanting as a function of metabolic state and BMI. Social Cognitive and Affective Neuroscience, 2014. 10(4): p. 561–568. doi: 10.1093/scan/nsu086 24948157 PMC4381239

[32] Croxson P.L., et al., Effort-based cost-benefit valuation and the human brain. J Neurosci, 2009. 29(14): p. 4531–41. doi: 10.1523/JNEUROSCI.4515-08.2009 19357278 PMC2954048

[33] Berridge K.C. and Robinson T.E., Liking, wanting, and the incentive-sensitization theory of addiction. Am Psychol, 2016. 71(8): p. 670–679. doi: 10.1037/amp0000059 27977239 PMC5171207

[34] Berridge K.C. and Kringelbach M.L., Pleasure Systems in the Brain. Neuron, 2015. 86(3): p. 646–664. doi: 10.1016/j.neuron.2015.02.018 25950633 PMC4425246

[35] Kühn S. and Gallinat J., The neural correlates of subjective pleasantness. NeuroImage, 2012. 61(1): p. 289–294. doi: 10.1016/j.neuroimage.2012.02.065 22406357

[36] Berridge K.C., Wanting and Liking: Observations from the Neuroscience and Psychology Laboratory. Inquiry (Oslo), 2009. 52(4): p. 378. doi: 10.1080/00201740903087359 20161627 PMC2813042

[37] Morales I. and Berridge K.C., ’Liking’ and ’wanting’ in eating and food reward: Brain mechanisms and clinical implications. Physiol Behav, 2020. 227: p. 113152. doi: 10.1016/j.physbeh.2020.113152 32846152 PMC7655589

[38] Salimpoor V.N., et al., Interactions Between the Nucleus Accumbens and Auditory Cortices Predict Music Reward Value. Science, 2013. 340(6129): p. 216–219. doi: 10.1126/science.1231059 23580531

[39] Winston J.S., et al., Relative Valuation of Pain in Human Orbitofrontal Cortex. The Journal of Neuroscience, 2014. 34(44): p. 14526–14535. doi: 10.1523/JNEUROSCI.1706-14.2014 25355207 PMC4212059

[40] Delgado M.R., et al., The role of the striatum in aversive learning and aversive prediction errors. Philos Trans R Soc Lond B Biol Sci, 2008. 363(1511): p. 3787–800. doi: 10.1098/rstb.2008.0161 18829426 PMC2607367

[41] Miller K.M., et al., How Should Consumers’ Willingness to Pay Be Measured? An Empirical Comparison of State-of-The-Art Approaches. Journal of Marketing Research, 2011. 48.

[42] Breidert C., Estimation of willingness-to-pay: Theory, measurement, application. Springer Science & Business Media., 2007.

[43] Breidert C., Hahsler M., and Reutterer T., A Review of Methods for Measuring Willingness-to-Pay. Innovative Marketing, 2015. 1.

[44] Wertenbroch K. and Skiera B., Measuring consumers’ willingness to pay at the point of purchase. Journal of marketing research, 2002. 39(2): p. 228–241.

[45] Acquisti A., Brandimarte L., and Loewenstein G., Privacy and human behavior in the age of information. Science, 2015. 347(6221): p. 509–514. doi: 10.1126/science.aaa1465 25635091

[46] Little J., Broadbent C.D., and Berrens R.P., Meta-analysis of the probability of disparity between actual and hypothetical valuation responses: Extension and preliminary new results. Western Economics Forum, 2012. 11(1837-2016-151799): p. 1–12.

[47] List J.A. and Gallet C.A., What Experimental Protocol Influence Disparities Between Actual and Hypothetical Stated Values? Environmental and Resource Economics, 2001. 20(3): p. 241–254.

[48] Murphy J.J., et al., A meta-analysis of hypothetical bias in stated preference valuation. Environmental and Resource Economics, 2005. 30: p. 313–325.

[49] Foster H. and Burrows J., Hypothetical bias: a new meta-analysis. 2017.

[50] Schmidt J. and Bijmolt T.H., Accurately measuring willingness to pay for consumer goods: a meta-analysis of the hypothetical bias. Journal of the Academy of Marketing Science, 2020. 48(3): p. 499–518.

[51] Kang M.J., et al., Hypothetical and real choice differentially activate common valuation areas. J Neurosci, 2011. 31(2): p. 461–8. doi: 10.1523/JNEUROSCI.1583-10.2011 21228156 PMC6623437

[52] Bray S., Shimojo S., and O’Doherty J.P., Human Medial Orbitofrontal Cortex Is Recruited During Experience of Imagined and Real Rewards. Journal of Neurophysiology, 2010. 103(5): p. 2506–2512. doi: 10.1152/jn.01030.2009 20200121

[53] Becker G.M., DeGroot M.H., and Marschak J., Measuring utility by a single‐response sequential method. Behavioral science, 1964. 9(3): p. 226–232. doi: 10.1002/bs.3830090304 5888778

[54] Padoa-Schioppa C., Neurobiology of economic choice: a good-based model. Annu Rev Neurosci, 2011. 34: p. 333–59. doi: 10.1146/annurev-neuro-061010-113648 21456961 PMC3273993

[55] Eickhoff S.B., et al., Activation likelihood estimation meta-analysis revisited. NeuroImage, 2012. 59(3): p. 2349–2361. doi: 10.1016/j.neuroimage.2011.09.017 21963913 PMC3254820

[56] Eickhoff S.B., et al., Coordinate-based activation likelihood estimation meta-analysis of neuroimaging data: A random-effects approach based on empirical estimates of spatial uncertainty. Human Brain Mapping, 2009. 30(9): p. 2907–2926. doi: 10.1002/hbm.20718 19172646 PMC2872071

[57] Peters J. and Buchel C., Neural representations of subjective reward value. Behav Brain Res, 2010. 213(2): p. 135–41. doi: 10.1016/j.bbr.2010.04.031 20420859

[58] Sescousse G., Li Y., and Dreher J.C., A common currency for the computation of motivational values in the human striatum. Soc Cogn Affect Neurosci, 2015. 10(4): p. 467–73. doi: 10.1093/scan/nsu074 24837478 PMC4381230

[59] Levy D.J. and Glimcher P.W., The root of all value: a neural common currency for choice. Curr Opin Neurobiol, 2012. 22(6): p. 1027–38. doi: 10.1016/j.conb.2012.06.001 22766486 PMC4093837

[60] Tyson-Carr J., et al., Neural correlates of economic value and valuation context: an event-related potential study. J Neurophysiol, 2018. 119(5): p. 1924–1933. doi: 10.1152/jn.00524.2017 29442556

[61] Grueschow M., et al., Automatic versus Choice-Dependent Value Representations in the Human Brain. Neuron, 2015. 85(4): p. 874–85. doi: 10.1016/j.neuron.2014.12.054 25640078

[62] Polanía R., et al., Neural Oscillations and Synchronization Differentially Support Evidence Accumulation in Perceptual and Value-Based Decision Making. Neuron, 2014. 82(3): p. 709–720. doi: 10.1016/j.neuron.2014.03.014 24811387

[63] Motoki K., Sugiura M., and Kawashima R., Common neural value representations of hedonic and utilitarian products in the ventral striatum: An fMRI study. Scientific Reports, 2019. 9(1): p. 15630. 31666605 10.1038/s41598-019-52159-9 PMC6821801

[64] Verdejo-Roman J., et al., Brain reward system’s alterations in response to food and monetary stimuli in overweight and obese individuals. Hum Brain Mapp, 2017. 38(2): p. 666–677. doi: 10.1002/hbm.23407 27659185 PMC6867019

[65] Liu X., et al., Common and distinct networks underlying reward valence and processing stages: a meta-analysis of functional neuroimaging studies. Neurosci Biobehav Rev, 2011. 35(5): p. 1219–36. doi: 10.1016/j.neubiorev.2010.12.012 21185861 PMC3395003

[66] Morelli S.A., Sacchet M.D., and Zaki J., Common and distinct neural correlates of personal and vicarious reward: A quantitative meta-analysis. Neuroimage, 2015. 112: p. 244–253. doi: 10.1016/j.neuroimage.2014.12.056 25554428 PMC4408229

[67] Clithero J.A. and Rangel A., Informatic parcellation of the network involved in the computation of subjective value. Social Cognitive and Affective Neuroscience, 2013. 9(9): p. 1289–1302. doi: 10.1093/scan/nst106 23887811 PMC4158359

[68] Evans A.C., et al. 3D statistical neuroanatomical models from 305 MRI volumes. in 1993 IEEE Conference Record Nuclear Science Symposium and Medical Imaging Conference. 1993.

[69] Talairach J., Co-planar stereotaxic atlas of the human brain-3-dimensional proportional system. An approach to cerebral imaging, 1988.

[70] Müller V.I., et al., Ten simple rules for neuroimaging meta-analysis. Neuroscience & Biobehavioral Reviews, 2018. 84: p. 151–161. doi: 10.1016/j.neubiorev.2017.11.012 29180258 PMC5918306

[71] Turkeltaub P.E., et al., Minimizing within‐experiment and within‐group effects in activation likelihood estimation meta‐analyses. Human brain mapping, 2012. 33(1): p. 1–13. doi: 10.1002/hbm.21186 21305667 PMC4791073

[72] Eickhoff, et al., Coordinate-based activation likelihood estimation meta-analysis of neuroimaging data: A random-effects approach based on empirical estimates of spatial uncertainty. Human Brain Mapping, 2009. 30(9): p. 2907–2926. doi: 10.1002/hbm.20718 19172646 PMC2872071

[73] Chib V.S., et al., Evidence for a common representation of decision values for dissimilar goods in human ventromedial prefrontal cortex. J Neurosci, 2009. 29(39): p. 12315–20. doi: 10.1523/JNEUROSCI.2575-09.2009 19793990 PMC6666137

[74] De Martino B., et al., Confidence in value-based choice. Nat Neurosci, 2013. 16(1): p. 105–10. doi: 10.1038/nn.3279 23222911 PMC3786394

[75] Enax L., et al., Nutrition labels influence value computation of food products in the ventromedial prefrontal cortex. Obesity (Silver Spring), 2015. 23(4): p. 786–92. doi: 10.1002/oby.21027 25755174

[76] Gluth S., et al., Effective Connectivity between Hippocampus and Ventromedial Prefrontal Cortex Controls Preferential Choices from Memory. Neuron, 2015. 86(4): p. 1078–1090. doi: 10.1016/j.neuron.2015.04.023 25996135

[77] Hare T.A., et al., Dissociating the role of the orbitofrontal cortex and the striatum in the computation of goal values and prediction errors. J Neurosci, 2008. 28(22): p. 5623–30. doi: 10.1523/JNEUROSCI.1309-08.2008 18509023 PMC6670807

[78] Hutcherson C.A., et al., Cognitive regulation during decision making shifts behavioral control between ventromedial and dorsolateral prefrontal value systems. J Neurosci, 2012. 32(39): p. 13543–54. doi: 10.1523/JNEUROSCI.6387-11.2012 23015444 PMC3689006

[79] Janowski V., Camerer C., and Rangel A., Empathic choice involves vmPFC value signals that are modulated by social processing implemented in IPL. Soc Cogn Affect Neurosci, 2013. 8(2): p. 201–8. doi: 10.1093/scan/nsr086 22349798 PMC3575723

[80] Linder N.S., et al., Organic labeling influences food valuation and choice. Neuroimage, 2010. 53(1): p. 215–20. doi: 10.1016/j.neuroimage.2010.05.077 20570738

[81] Mackey S., et al., Greater preference consistency during the Willingness-to-Pay task is related to higher resting state connectivity between the ventromedial prefrontal cortex and the ventral striatum. Brain Imaging Behav, 2016. 10(3): p. 730–8. doi: 10.1007/s11682-015-9435-z 26271206 PMC4753147

[82] McNamee D., Rangel A., and O’doherty J.P., Category-dependent and category-independent goal-value codes in human ventromedial prefrontal cortex. Nat Neurosci, 2013. 16(4): p. 479–85. doi: 10.1038/nn.3337 23416449 PMC3665508

[83] Medic N., et al., Dopamine modulates the neural representation of subjective value of food in hungry subjects. J Neurosci, 2014. 34(50): p. 16856–64. doi: 10.1523/JNEUROSCI.2051-14.2014 25505337 PMC4261106

[84] Merchant J.S., et al., Neural Substrates of Food Valuation and Its Relationship With BMI and Healthy Eating in Higher BMI Individuals. Front Behav Neurosci, 2020. 14: p. 578676. doi: 10.3389/fnbeh.2020.578676 33343310 PMC7746820

[85] Motoki K., Sugiura M., and Kawashima R., Common neural value representations of hedonic and utilitarian products in the ventral stratum: An fMRI study. Sci Rep, 2019. 9(1): p. 15630. doi: 10.1038/s41598-019-52159-9 31666605 PMC6821801

[86] Plassmann H., O’Doherty J.P., and Rangel A., Appetitive and aversive goal values are encoded in the medial orbitofrontal cortex at the time of decision making. J Neurosci, 2010. 30(32): p. 10799–808. doi: 10.1523/JNEUROSCI.0788-10.2010 20702709 PMC6634706

[87] Rihm J.S., et al., Sleep Deprivation Selectively Upregulates an Amygdala-Hypothalamic Circuit Involved in Food Reward. J Neurosci, 2019. 39(5): p. 888–899. doi: 10.1523/JNEUROSCI.0250-18.2018 30559151 PMC6382977

[88] Seak L.C.U., et al., Single-Dimensional Human Brain Signals for Two-Dimensional Economic Choice Options. The Journal of Neuroscience, 2021. 41(13): p. 3000–3013. doi: 10.1523/JNEUROSCI.1555-20.2020 33568490 PMC8018883

[89] Setton R., Fisher G., and Spreng R.N., Mind the gap: Congruence between present and future motivational states shapes prospective decisions. Neuropsychologia, 2019. 132: p. 107130. doi: 10.1016/j.neuropsychologia.2019.107130 31276683 PMC6702072

[90] Tang D.W., Fellows L.K., and Dagher A., Behavioral and neural valuation of foods is driven by implicit knowledge of caloric content. Psychol Sci, 2014. 25(12): p. 2168–76. doi: 10.1177/0956797614552081 25304885

[91] Verdejo-Roman J., et al., Independent functional connectivity networks underpin food and monetary reward sensitivity in excess weight. Neuroimage, 2017. 146: p. 293–300. doi: 10.1016/j.neuroimage.2016.11.011 27856313

[92] Waskow S., et al., Pay What You Want! A Pilot Study on Neural Correlates of Voluntary Payments for Music. Front Psychol, 2016. 7: p. 1023. doi: 10.3389/fpsyg.2016.01023 27458416 PMC4933710

[93] Zangemeister L., Grabenhorst F., and Schultz W., Neural activity in human ventromedial prefrontal cortex reflecting the intention to save reward. Soc Cogn Affect Neurosci, 2019. 14(12): p. 1255–1261. doi: 10.1093/scan/nsaa013 31993656 PMC7137725

[94] Maris E. and Oostenveld R., Nonparametric statistical testing of EEG- and MEG-data. Journal of Neuroscience Methods, 2007. 164(1): p. 177–190. doi: 10.1016/j.jneumeth.2007.03.024 17517438

[95] Eickhoff, et al., Behavior, sensitivity, and power of activation likelihood estimation characterized by massive empirical simulation. Neuroimage, 2016. 137: p. 70–85. doi: 10.1016/j.neuroimage.2016.04.072 27179606 PMC4981641

[96] Maris E. and Oostenveld R., Nonparametric statistical testing of EEG- and MEG-data. Journal of Neuroscience Methods, 2007. 164(1): p. 177–190.73. Eickhoff, et al., Behavior, sensitivity, and power of activation likelihood estimation characterized by massive empirical simulation. Neuroimage, 2016. 137: p. 70–85. doi: 10.1016/j.jneumeth.2007.03.024 17517438

[97] Fallon N., Roberts C., and Stancak A., Shared and distinct functional networks for empathy and pain processing: a systematic review and meta-analysis of fMRI studies. Social Cognitive and Affective Neuroscience, 2020. 15(7): p. 709–723. doi: 10.1093/scan/nsaa090 32608498 PMC7511882

[98] Papitto G., Friederici A.D., and Zaccarella E., The topographical organization of motor processing: An ALE meta-analysis on six action domains and the relevance of Broca’s region. NeuroImage, 2020. 206: p. 116321. doi: 10.1016/j.neuroimage.2019.116321 31678500

[99] Hoffman P. and Morcom A.M., Age-related changes in the neural networks supporting semantic cognition: A meta-analysis of 47 functional neuroimaging studies. Neuroscience & Biobehavioral Reviews, 2018. 84: p. 134–150. doi: 10.1016/j.neubiorev.2017.11.010 29183684

[100] Gan X., et al., Common and distinct neurofunctional representations of core and social disgust in the brain: Coordinate-based and network meta-analyses. Neuroscience & Biobehavioral Reviews, 2022. 135: p. 104553. doi: 10.1016/j.neubiorev.2022.104553 35122784

[101] Rothstein H.R., Sutton A.J., and Borenstein M., Publication bias in meta-analysis. Publication bias in meta-analysis: Prevention, assessment and adjustments, 2005: p. 1–7.

[102] Rosenthal R., The file drawer problem and tolerance for null results. Psychological bulletin, 1979. 86(3): p. 638.

[103] Acar F., et al., Assessing robustness against potential publication bias in Activation Likelihood Estimation (ALE) meta-analyses for fMRI. PloS one, 2018. 13(11): p. e0208177. doi: 10.1371/journal.pone.0208177 30500854 PMC6267999

[104] Samartsidis P., et al., Estimating the prevalence of missing experiments in a neuroimaging meta‐analysis. Research synthesis methods, 2020. 11(6): p. 866–883. doi: 10.1002/jrsm.1448 32860642 PMC7780291

[105] Pando-Naude V., et al., An ALE meta-analytic review of top-down and bottom-up processing of music in the brain. Scientific Reports, 2021. 11(1): p. 20813. doi: 10.1038/s41598-021-00139-3 34675231 PMC8531391

[106] Diekhof E.K., et al., The role of the human ventral striatum and the medial orbitofrontal cortex in the representation of reward magnitude—an activation likelihood estimation meta-analysis of neuroimaging studies of passive reward expectancy and outcome processing. Neuropsychologia, 2012. 50(7): p. 1252–66. doi: 10.1016/j.neuropsychologia.2012.02.007 22366111

[107] Strait C.E., Blanchard T.C., and Hayden B.Y., Reward Value Comparison via Mutual Inhibition in Ventromedial Prefrontal Cortex. Neuron, 2014. 82(6): p. 1357–1366. doi: 10.1016/j.neuron.2014.04.032 24881835 PMC4086796

[108] Henri-Bhargava A., Simioni A., and Fellows L.K., Ventromedial frontal lobe damage disrupts the accuracy, but not the speed, of value-based preference judgments. Neuropsychologia, 2012. 50(7): p. 1536–1542. doi: 10.1016/j.neuropsychologia.2012.03.006 22433288

[109] Fellows L.K., Orbitofrontal contributions to value-based decision making: evidence from humans with frontal lobe damage. Ann N Y Acad Sci, 2011. 1239: p. 51–8. doi: 10.1111/j.1749-6632.2011.06229.x 22145875

[110] Tremblay L. and Schultz W., Relative reward preference in primate orbitofrontal cortex. Nature, 1999. 398(6729): p. 704. doi: 10.1038/19525 10227292

[111] Lopatina N., et al., Medial Orbitofrontal Neurons Preferentially Signal Cues Predicting Changes in Reward during Unblocking. J Neurosci, 2016. 36(32): p. 8416–24. doi: 10.1523/JNEUROSCI.1101-16.2016 27511013 PMC4978801

[112] Vogt B.A., Pain and emotion interactions in subregions of the cingulate gyrus. Nature Reviews Neuroscience, 2005. 6(7): p. 533–544. doi: 10.1038/nrn1704 15995724 PMC2659949

[113] Phan K.L., et al., Functional Neuroanatomy of Emotion: A Meta-Analysis of Emotion Activation Studies in PET and fMRI. NeuroImage, 2002. 16(2): p. 331–348. doi: 10.1006/nimg.2002.1087 12030820

[114] Stevens F.L., Hurley R.A., and Taber K.H., Anterior Cingulate Cortex: Unique Role in Cognition and Emotion. The Journal of Neuropsychiatry and Clinical Neurosciences, 2011. 23(2): p. 121–125. doi: 10.1176/jnp.23.2.jnp121 21677237

[115] Blair K.S., et al., Dissociable roles of ventromedial prefrontal cortex (vmPFC) and rostral anterior cingulate cortex (rACC) in value representation and optimistic bias. NeuroImage, 2013. 78: p. 103–110. doi: 10.1016/j.neuroimage.2013.03.063 23567883 PMC3686504

[116] Beckmann M., Johansen-Berg H., and Rushworth M.F.S., Connectivity-Based Parcellation of Human Cingulate Cortex and Its Relation to Functional Specialization. The Journal of Neuroscience, 2009. 29(4): p. 1175. doi: 10.1523/JNEUROSCI.3328-08.2009 19176826 PMC6665147

[117] Grabenhorst F. and Rolls E.T., Value, pleasure and choice in the ventral prefrontal cortex. Trends in Cognitive Sciences, 2011. 15(2): p. 56–67. doi: 10.1016/j.tics.2010.12.004 21216655

[118] Cai X. and Padoa-Schioppa C., Neuronal Encoding of Subjective Value in Dorsal and Ventral Anterior Cingulate Cortex. The Journal of Neuroscience, 2012. 32(11): p. 3791–3808. doi: 10.1523/JNEUROSCI.3864-11.2012 22423100 PMC3319456

[119] Kennerley S.W., et al., Neurons in the frontal lobe encode the value of multiple decision variables. Journal of Cognitive Neuroscience, 2009. 21(6): p. 1162–1178. doi: 10.1162/jocn.2009.21100 18752411 PMC2715848

[120] Hosokawa T., et al., Single-Neuron Mechanisms Underlying Cost-Benefit Analysis in Frontal Cortex. The Journal of Neuroscience, 2013. 33(44): p. 17385. doi: 10.1523/JNEUROSCI.2221-13.2013 24174671 PMC3812506

[121] Strait C.E., Sleezer B., and Hayden B., Signatures of Value Comparison in Ventral Striatum Neurons. PLoS Biol, 2015. 13(6): p. e1002173. doi: 10.1371/journal.pbio.1002173 26086735 PMC4472856

[122] Filimon F., et al., The ventral striatum dissociates information expectation, reward anticipation, and reward receipt. Proceedings of the National Academy of Sciences, 2020. 117(26): p. 15200. doi: 10.1073/pnas.1911778117 32527855 PMC7334472

[123] Rutledge R.B., et al., Testing the Reward Prediction Error Hypothesis with an Axiomatic Model. The Journal of Neuroscience, 2010. 30(40): p. 13525. doi: 10.1523/JNEUROSCI.1747-10.2010 20926678 PMC2957369

[124] Camille N., et al., Ventromedial Frontal Lobe Damage Disrupts Value Maximization in Humans. The Journal of Neuroscience, 2011. 31(20): p. 7527. doi: 10.1523/JNEUROSCI.6527-10.2011 21593337 PMC3122333

[125] Lim S.L., O’Doherty J.P., and Rangel A., The decision value computations in the vmPFC and striatum use a relative value code that is guided by visual attention. J Neurosci, 2011. 31(37): p. 13214–23. doi: 10.1523/JNEUROSCI.1246-11.2011 21917804 PMC6623246

[126] Deserno L., et al., Ventral striatal dopamine reflects behavioral and neural signatures of model-based control during sequential decision making. Proc Natl Acad Sci U S A, 2015. 112(5): p. 1595–600. doi: 10.1073/pnas.1417219112 25605941 PMC4321318

[127] Yacubian J., et al., Subregions of the ventral striatum show preferential coding of reward magnitude and probability. NeuroImage, 2007. 38(3): p. 557–563. doi: 10.1016/j.neuroimage.2007.08.007 17889562

[128] Tom S.M., et al., The neural basis of loss aversion in decision-making under risk. Science, 2007. 315(5811): p. 515–518. doi: 10.1126/science.1134239 17255512

[129] Cromwell H.C. and Schultz W., Effects of Expectations for Different Reward Magnitudes on Neuronal Activity in Primate Striatum. Journal of Neurophysiology, 2003. 89(5): p. 2823–2838. doi: 10.1152/jn.01014.2002 12611937

[130] Naqvi N.H. and Bechara A., The hidden island of addiction: the insula. Trends in neurosciences, 2009. 32(1): p. 56–67. doi: 10.1016/j.tins.2008.09.009 18986715 PMC3698860

[131] Craig A.D., How do you feel—now? The anterior insula and human awareness. Nature reviews neuroscience, 2009. 10(1): p. 59–70. doi: 10.1038/nrn2555 19096369

[132] Bechara A. and Damasio A.R., The somatic marker hypothesis: A neural theory of economic decision. Games and economic behavior, 2005. 52(2): p. 336–372.

[133] Critchley H.D., Neural mechanisms of autonomic, affective, and cognitive integration. Journal of comparative neurology, 2005. 493(1): p. 154–166. doi: 10.1002/cne.20749 16254997

[134] Quartz S.R., Reason, emotion and decision-making: risk and reward computation with feeling. Trends in cognitive sciences, 2009. 13(5): p. 209–215. doi: 10.1016/j.tics.2009.02.003 19362037

[135] Namkung H., Kim S.-H., and Sawa A., The insula: an underestimated brain area in clinical neuroscience, psychiatry, and neurology. Trends in neurosciences, 2017. 40(4): p. 200–207. doi: 10.1016/j.tins.2017.02.002 28314446 PMC5538352

[136] Paulus M.P. and Stein M.B., An insular view of anxiety. Biological psychiatry, 2006. 60(4): p. 383–387. doi: 10.1016/j.biopsych.2006.03.042 16780813

[137] Brown S., et al., Naturalizing aesthetics: brain areas for aesthetic appraisal across sensory modalities. Neuroimage, 2011. 58(1): p. 250–258. doi: 10.1016/j.neuroimage.2011.06.012 21699987 PMC8005853

[138] Platt M.L. and Huettel S.A., Risky business: the neuroeconomics of decision making under uncertainty. Nat Neurosci, 2008. 11(4): p. 398–403. doi: 10.1038/nn2062 18368046 PMC3065064

[139] Mohr P.N., Biele G., and Heekeren H.R., Neural processing of risk. J Neurosci, 2010. 30(19): p. 6613–9. doi: 10.1523/JNEUROSCI.0003-10.2010 20463224 PMC6632558

[140] Loued-Khenissi L., et al., Anterior insula reflects surprise in value-based decision-making and perception. NeuroImage, 2020. 210: p. 116549. doi: 10.1016/j.neuroimage.2020.116549 31954844

[141] van den Bos W., et al., The value of victory: social origins of the winner’s curse in common value auctions. Judgm Decis Mak, 2008. 3(7): p. 483–492. 20305741 PMC2841440

[142] Kagel J.H., et al., First‐price common value auctions: bidder behavior and the “Winner’s Curse”. Economic Inquiry, 1989. 27(2): p. 241–258.

[143] Staudinger M.R., Erk S., and Walter H., Dorsolateral Prefrontal Cortex Modulates Striatal Reward Encoding during Reappraisal of Reward Anticipation. Cerebral Cortex, 2011. 21(11): p. 2578–2588. doi: 10.1093/cercor/bhr041 21459835

[144] Aron A.R., et al., Stop-signal inhibition disrupted by damage to right inferior frontal gyrus in humans. Nature Neuroscience, 2003. 6(2): p. 115–116. doi: 10.1038/nn1003 12536210

[145] Hampshire A., et al., The role of the right inferior frontal gyrus: inhibition and attentional control. NeuroImage, 2010. 50(3): p. 1313–1319. doi: 10.1016/j.neuroimage.2009.12.109 20056157 PMC2845804

[146] Aron A.R., Robbins T.W., and Poldrack R.A., Inhibition and the right inferior frontal cortex: one decade on. Trends Cogn Sci, 2014. 18(4): p. 177–85. doi: 10.1016/j.tics.2013.12.003 24440116

[147] Aron A.R., Robbins T.W., and Poldrack R.A., Inhibition and the right inferior frontal cortex. Trends Cogn Sci, 2004. 8(4): p. 170–7. doi: 10.1016/j.tics.2004.02.010 15050513

[148] Hare T.A., Camerer C.F., and Rangel A., Self-Control in Decision-Making Involves Modulation of the vmPFC Valuation System. Science, 2009. 324(5927): p. 646–648. doi: 10.1126/science.1168450 19407204

[149] McClure S.M., et al., Separate Neural Systems Value Immediate and Delayed Monetary Rewards. Science, 2004. 306(5695): p. 503–507. doi: 10.1126/science.1100907 15486304

[150] Chen F., et al., Increased BOLD Signals in dlPFC Is Associated With Stronger Self-Control in Food-Related Decision-Making. Frontiers in Psychiatry, 2018. 9. doi: 10.3389/fpsyt.2018.00689 30618869 PMC6306453

[151] Huber R.E., Klucharev V., and Rieskamp J., Neural correlates of informational cascades: brain mechanisms of social influence on belief updating. Social Cognitive and Affective Neuroscience, 2014. 10(4): p. 589–597. doi: 10.1093/scan/nsu090 24974396 PMC4381243

[152] Jarcho J.M., Berkman E.T., and Lieberman M.D., The neural basis of rationalization: cognitive dissonance reduction during decision-making. Social Cognitive and Affective Neuroscience, 2010. 6(4): p. 460–467. doi: 10.1093/scan/nsq054 20621961 PMC3150852

[153] McClure S.M., et al., Time Discounting for Primary Rewards. The Journal of Neuroscience, 2007. 27(21): p. 5796–5804. doi: 10.1523/JNEUROSCI.4246-06.2007 17522323 PMC6672764

[154] Huettel S.A., et al., Neural Signatures of Economic Preferences for Risk and Ambiguity. Neuron, 2006. 49(5): p. 765–775. doi: 10.1016/j.neuron.2006.01.024 16504951

[155] Raggetti G., et al., Neural Correlates of Direct Access Trading in a Real Stock Market: An fMRI Investigation. Front Neurosci, 2017. 11: p. 536. doi: 10.3389/fnins.2017.00536 29033782 PMC5626870

[156] Gottfried J.A., O’Doherty J., and Dolan R.J., Encoding predictive reward value in human amygdala and orbitofrontal cortex. Science, 2003. 301(5636): p. 1104–7. doi: 10.1126/science.1087919 12934011

[157] Kringelbach M.L., et al., Activation of the human orbitofrontal cortex to a liquid food stimulus is correlated with its subjective pleasantness. Cerebral cortex, 2003. 13(10): p. 1064–1071. doi: 10.1093/cercor/13.10.1064 12967923

[158] Karni E. and Safra Z., " Preference reversal" and the observability of preferences by experimental methods. Econometrica: Journal of the Econometric Society, 1987: p. 675–685.

[159] Horowitz J.K., The Becker-DeGroot-Marschak mechanism is not necessarily incentive compatible, even for non-random goods. Economics Letters, 2006. 93(1): p. 6–11.

[160] Newton-Fenner A., et al., A comparison of reward processing during Becker–DeGroot–Marschak *and Vickrey auctions*: *An ERP study*. Psychophysiology, 2023. n/a(n/a): p. e14313. 10.1111/psyp.14313 PMC10909440 37076995

