# Supplementary material for: Economic value in the Brain: A meta-analysis of willingness-to-pay using the Becker-DeGroot-Marschak auction
Source: PLoS One. 2023 Jul 10;18(7):e0286969. doi: 10.1371/journal.pone.0286969 (PMC10332630; doi:10.1371/journal.pone.0286969)
Supplement: S1 File — (DOCX) [file pone.0286969.s001.docx]

**S1. Formal proof of the Dominant Strategy in BDM Auctions.**

*Scenario:*

*Suppose there is a single player in a BDM auction for a single item. The player – denoted i — is given an endowment and asked to submit a bid b_i_. Simultaneously, a random number b_r_ is generated.*

*The allocation rule is:*

*if b_i_ > b_r_: player i buys the item at price b_r_;*

*if b_i_ < b_r_: player i keeps the endowment;*

*if b_i_ = b_r_: either (i) or (ii) is implemented with equal probability.*

*Strategy:*

*Suppose that player i’s valuation of an item is v_i_, and the auction has a reserve price R. If b_i_ > b_r_, the payoff for player i is v_i_ – b_r_. If b_i_ < b_r_, the payoff for player i is 0.*

*a. When v_i_ < R*

*Player i does not want to win the auction because they would have to pay at least R, which will cause them to make a loss. Hence, bidding b_i_ = v_i_ is optimal since they will end up not winning the auction.*

*b. When v_i_ >= R*

*Player i has three options, b_i_ > v_i_, b_i_ < v_i_ and b_i_=v_i_*

*Consider a deviation b_i_ > v_i_:*

*It is possible that b_i_ > b_r_ > v_i_, in which case player i will win the auction with a negative surplus v_i_ - b_r_.*

*Consider a deviation b_i_ < v_i_:*

*It is possible that b_i_ < b_r_ < v_i_, in which case player i loses the auction with a payoff of 0, whereas they could have won it with a positive payoff of v_i_ - b_r_ if they had bid b_i_ = v_i_.*

*Hence bidding b_i_ = v_i_ is the weakly dominant strategy.*

*The payoff strategy of the BDM is as follows:*

$$\left\{ \begin{aligned} \nu_{i} -{max}_{r\neq i}b_{r} ifb_{i} >{max}_{r\neq i}b_{r} \\ 0 Otherwise \end{aligned} \right.$$
